# Scoping Review of Experimental and Clinical Evidence and Its Influence on Development of the Suction Ureteral Access Sheath

**DOI:** 10.3390/diagnostics14101034

**Published:** 2024-05-16

**Authors:** Steffi Kar Kei Yuen, Olivier Traxer, Marcelo Langer Wroclawski, Nariman Gadzhiev, Chu Ann Chai, Ee Jean Lim, Carlo Giulioni, Virgilio De Stefano, Carlotta Nedbal, Martina Maggi, Kemal Sarica, Daniele Castellani, Bhaskar Somani, Vineet Gauhar

**Affiliations:** 1 SH Ho Urology Centre, Department of Surgery, The Chinese University of Hong Kong, Hong Kong, China; 2 Department of Urology AP-HP, Sorbonne University, Tenon Hospital, 75020 Paris, France; olivier.traxer@aphp.fr; 3 Hospital Israelita Albert Einstein, São Paulo 05652-900, Brazil; urologia.marcelo@gmail.com; 4 BP—A Beneficência Portuguesa de São Paulo, São Paulo 01451-010, Brazil; 5 Urology Department, Saint-Petersburg State University Hospital, 197342 St. Petersburg, Russia; nariman.gadjiev@gmail.com; 6 Urology Unit, Surgery Department, University Malaya Medical Center, Petaling Jaya 50603, Malaysia; chaichuann@yahoo.com; 7 Department of Urology, Singapore General Hospital, Singapore 169608, Singapore; eejeanlim@gmail.com; 8 Department of Urology, Casa di Cura Villa Igea, 60127 Ancona, Italy; carlo.giulioni9@gmail.com; 9 Urology Unit, Azienda Ospedaliero Universitaria delle Marche, 60121 Ancona, Italy; virgilio.destefano@gmail.com (V.D.S.); carlottanedbal@gmail.com (C.N.); castellanidaniele@gmail.com (D.C.); 10 Urology Unit, ASST Fatebenefratelli Sacco, 20131 Milano, Italy; 11 Department of Maternal Infant and Urological Sciences, Sapienza University of Rome, 00185 Rome, Italy; martina.maggi@uniroma1.it; 12 Department of Urology, Biruni University, 34015 Istanbul, Turkey; saricakemal@gmail.com; 13 Department of Urology, University Hospitals Southampton, NHS Trust, Southampton SO16 6YD, UK; bhaskarsomani@yahoo.com; 14 Department of Urology, Ng Teng Fong General Hospital, Singapore 126817, Singapore

**Keywords:** RIRS, retrograde intrarenal surgery, flexible ureteroscopy, ureteral access sheath, suction, FANS, flexible and navigable ureteral access sheath

## Abstract

The ureteral access sheath (UAS) has been a boon and a bane in flexible ureteroscopy (FURS), with its merits and demerits well established. Its design and dimensions were instrumental in reshaping the way flexible scopes were used and were key adjuncts to establishing retrograde intrarenal surgery (RIRS) as a standard of care in the endourological management of renal stones. With the ever-changing landscape of RIRS over the decades shaped by technological advancements in lasers and flexible scopes, the UAS has also continuously evolved. The utility of suction in endourology has recently changed the way RIRS is performed and is a game changer for FURS outcomes. With strong clinical and experimental evidence to support its use, the UAS has undergone a transformative change in the recent past, with its ability to monitor intrarenal pressure and provide a superior vacuum-cleaner effect that improves the trifecta of RIRS, namely an improved single-stage stone-free rate (SFR), minimise complications, and reduce reinterventions. Our comprehensive review outlines the key clinical and experimental evidence and traces the developments that were key to modifying the traditional UAS into a flexible and navigable suction ureteric access sheath (FANS) and highlights how the design and modifications, in turn, influence the ability to push the boundaries of RIRS.

## 1. Introduction

### 1.1. Historical Role of Ureteral Access Sheaths in Retrograde Intrarenal Surgery

The use of ureteral access sheath (UAS) started in the 1970s, when the first prototypes of the UAS were developed [1]. Ideated to overcome the issues related to multiple withdrawals and reinsertion of the flexible ureteroscope in the upper urinary tract, as well as the problem caused by the buckling of the scope into the bladder, the use of the UAS was rapidly popularized worldwide. The International Alliance of Urolithiasis Guidelines [2] recommend that the placement of a UAS may facilitate retrograde intrarenal surgery (RIRS).

While providing a working channel for the easy insertion of instruments into the renal collecting system, a UAS also helps in improving visibility during RIRS, reducing the intrarenal pressure (IRP) and, thus, minimising the risk of parenchymal damage and sepsis [3,4,5,6,7,8]. UASs have been correlated with shorter operative times [9], higher stone-free rates (SFR) in patients presenting with multiple renal calculi or calculi > 5 mm, and lower incidence of complications [10]. The benefit of a UAS is also noted when adopting the high-power Holmium:Yttrium-Aluminium-Garnet (Ho:YAG), the Thulium Fibre Laser(TFL), and the Thulium:YAG laser, particularly in scenarios such as bilateral RIRS and in paediatric patients [11,12,13]. The associated costs and risks of using a UAS could be potentially justified by the aforementioned advantages [4,14]. Even in light of the undeniable benefits, care must be taken during the placement and use of a UAS, as it can cause possible trauma to the ureter, perforation, or iatrogenic strictures [15]. An important consideration highlighted by Kaler et al. is the amount of buckling force deployed for placement. The authors reported that serious ureteral damage routinely occurred when forces exceeded 8.1 N and, hence, serial dilation of a non-stented ureter, which may allow safe passage at higher deployment forces, as much as 5.56 N. Whilst clinically difficult to measure, this does provide a clear message that deployment of a UAS must be smooth, traumatic with the least use of force, and never done against resistance [16].

### 1.2. The Utility of UAS and Recommendations in Guidelines

With all guidelines embracing its use [17,18,19,20], the utility of UAS is now an accepted part of routine practice in RIRS. The European Association of Urology (EAU) guidelines on urolithiasis state that the use of the UAS is safe and can be useful for large and multiple renal stones or if a long procedural time is expected [19]. The positive role of the UAS in RIRS is also discussed in the American Urological Association (AUA) guidelines, where the use of UAS is linked to improved outcomes and reduced risks [20]. However, there is no specific recommendation on the criteria for patient selection for UAS insertion.

Nowadays, different sizes and measures of UASs are available on the market, providing the urologist with plenty of choices according to the patient’s anatomical characteristics and ureteral compliance. It is important to remember that the presence of a significant gap >1.8 Ch (0.6 mm) between the inner channel of the UAS and the outer margin of the operative instrument helps the flow of irrigation fluids, hence increasing the benefits of the UAS placement [21]. Shi J et al. [22] proposed that, to maintain safe IRP, the combination of FURS and UAS should maintain a basic rule, which is the ratio of the endoscope–sheath diameter of ≤0.75. This was also confirmed by Li Fang et al. [23].

The main concern of placement of the UAS is the possible increased risk of ureteral wall ischaemia and injury to the mucosal or muscular layers of the ureter, theoretically increasing the risk of ureteral strictures and, hence, the need for postoperative stent placement [24,25]. This is why most surgeons prefer to use smaller diameter UASs, which often do not need preoperative stenting. Yet, some argue that increasing the size improves surgical efficiency without increasing complications [3].

There is no consensus on the optimal sheath size, but as scopes get iniaturized, it allows for the usage of a smaller calibre UAS. In a recent study, it was shown that, as the UAS calibration increased, the SFR also increased and that calibration increase itself was an independent predictive factor for stone-free status, with the highest predictive value seen with a 10–12 Fr UAS [26].

### 1.3. Role of Suction in Endourology and RIRS

In endourology, suction plays a pivotal role in facilitating the removal of debris, irrigation fluids, and stone fragments during various minimally invasive procedures. The validity of employing these instruments has been increasingly delineated, particularly in percutaneous nephrolithotomy (PCNL), as per the EAU guidelines [19]. A recent systematic review has introduced various suction devices for RIRS, including those deployed through the UAS or directly attached to the scope, offering control of IRP [27]. Evidence from this review underscores the effectiveness of these devices in RIRS. In terms of procedural safety, the regulation of IRP and the ability to enhance irrigation flow during the procedure help mitigate the risks associated with fluid-pump mechanisms, which may result in mucosal injury within the collecting system and the reabsorption of irrigation fluid containing bacteria and endotoxins caused by pyelovenous backflow [28]. Additionally, thorough cleansing of the pelvocalyceal system from debris and residual fragments appears linked to a reduced incidence of postoperative infectious complications [29]. It may be attributed to decreased reliance on accessory instruments, such as baskets, and manipulation of the scope [30].

Suction devices have also demonstrated improved efficacy in RIRS, leading to reduced operative times and increased SFR [27,31]. These outcomes can be justified by several factors. First, the removal of debris enhances intraoperative visibility, mitigating the “snow-globe effect” and facilitating the identification of fragments hidden beneath it [32]. Moreover, this accessory obviates the need for fragment extraction, a time-consuming process impractical for fragments smaller than 2 mm [33]. Lastly, debris aspiration also diminishes the incidence of steinstrasse. With advancements in RIRS technology, there is a growing inclination towards the endoscopic management of progressively larger renal calculi [34]. The introduction of this new technology could prove even more beneficial in challenging cases involving voluminous calculi. Therefore, the utilization of suction devices in RIRS has led to significant improvements in patient outcomes, enhancing SFR, and contributing to a decreased need for secondary interventions. The suction ureteral access sheath (SUAS) is the first prototype in the evolution of such technical advancement, followed by the flexible and navigable ureteral access sheath (FANS) with the use of suction in ureteroscopy. In our study, we highlight how such improvements in the UAS design have improved RIRS and this, in turn, has led to further significant changes in the design and properties of the SUAS.

## 2. Material and Methods

**Figure 1:**
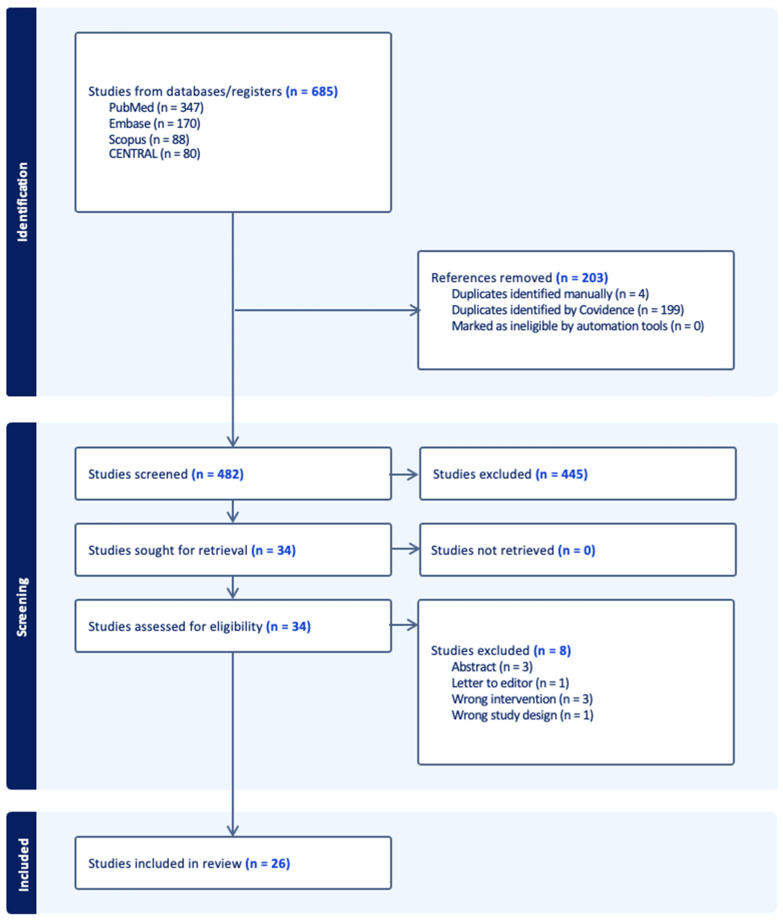
PRISMA diagram.

The publication search was conducted from inception to 14 February 2024 in several databases, including PubMed, Medline, Embase, and Scopus via Boolean operators with the use of the following terms: ‘flexible’, ‘ureteral access sheath’, ‘navigable’, ‘bendable’, ‘suction’, ‘vacuum’, ‘ureteroscopy’, and ‘retrograde intrarenal surgery’ to find studies on the flexible and navigable ureteral access sheath with the use of suction in ureteroscopy (Figure 1). Duplicates were removed from screening, and reviews, editorials, and case reports were excluded from the full-text eligibility assessment. Only articles in English were included. The literature analysis was realized and confirmed for final selection independently by two authors (S.K.K.Y. and V.G.). According to the substantial degree of heterogeneity among the included studies, in terms of both design and outcomes, a systematic review or mathematical summary of the results of selected studies was not performed. Instead, we identified papers to perform a narrative review, with the focus on the use of different ureteral access sheaths, including flexible and navigable ureteral access sheaths with the use of suction in ureteroscopy. This scoping review was conducted in compliance with PRISMA guidelines.

## 3. Results

Of the 26 studies selected, 4 were experimental studies, and the rest were clinical studies. Of all 22 clinical studies, 7 were related to SUAS, 5 reported SUAS with a pressure sensing mechanism, and 10 were on FANS.

### 3.1. Results of Review of Experimental Studies (Table 1)

From 2016 to 2022, four experimental studies have been conducted to investigate various suction and irrigation mechanisms of RIRS. Two ex vivo studies and two in vitro studies were carried out, utilising a total of 56 porcine kidney specimens. These studies aimed to enhance the understanding of the RIRS techniques, particularly focusing on minimising IRP and optimising SFR.

Chen et al. conducted an ex vivo study involving 20 porcine kidneys to compare the safety and efficacy of FANS versus traditional UAS, incorporating a pressure sensor positioned in the renal calyx through renal puncture [35]. The findings indicated that FANS led to significantly lower IRP while achieving an SFR of 70% compared to the UAS group. Notably, the traditional UAS group exhibited a clinically significant residual stone volume (92.5 vs. 33.7 mm^3^), emphasising the superiority of FANS in this aspect.

Zhu et al. performed an in vivo study on 18 porcine kidneys, evaluating the utility of a pressure-control device for monitoring and regulating IRP at the renal pelvis and calyces across various irrigation flow rates [36]. Their results demonstrated that the renal pelvis and calyces pressures remained consistent across different irrigation flow rates, facilitated by the implementation of a pressure-control irrigation and suctioning device.

Wang et al. conducted another ex vivo study utilising 12 porcine kidneys, aiming to establish the superiority of SUAS over UAS in maintaining lower IRP [37]. By deploying a 6 Fr pressure-sensor catheter in different calyces, they revealed that SUAS effectively maintained lower IRP levels, particularly evident at higher irrigation rates exceeding 100 cc/min.

Finally, Ostegar et al. investigated optimal SUAS settings through an in vivo study involving six porcine kidneys, utilising IRP monitoring with a Swan–Ganz catheter [38]. Their findings indicated that, while SUAS implementation reduced the mean IRP during RIRS, excessive suction with high vacuum levels (>200 mmHg) posed a risk of outflow-tract collapse, emphasising the importance of cautious suction management.

**Table 1:** Experimental Studies.

Author	Year	Country	Study Type	Sample Size	Suction Modality	Outcome
Zhu et al. [36]	2016	China	in vivo	9 live pigs (18 kidneys units)	SUAS with IRP measure and suctioning/working channel	IRPs were similar to those recorded by the invasive blood pressure monitor. IRP from the renal pelvic outlet were similar to those from the upper calyceal area
Chen et al. [35]	2022	China	ex vivo	20 porcine cadaveric kidneys	12/14 Fr FANS, 46 cm vs. UAS Pressure sensor was placed in the renal calix by renal puncture	FANS vs. UAS group: Operative time 44.2 vs. 39.7 *p* = 0.1 Residual stone volume 33.7 vs. 92.5 *p* = 0.017 Stone clearance 98.5% vs. 95.9% *p* = 0.017 Complete SFR: 70% FANS
Wang et al. [37]	2022	China	ex vivo	12 adult porcine fresh harvested kidneys	12/14 Fr SUAS vs. UAS 6 Fr pressure monitor catheters via renal puncture	SUAS maintains lower IRP than UAS under same parameters. Both SUAS and UAS can be used when irrigation is ≤50 cc/min; SUAS showed clear advantages over UAS in maintaining lower pressure when irrigation rate is ≥100 cc/min.
Ostergar et al. [38]	2022	USA	in vivo	3 female porcine cadaveric kidneys	SUAS connected to wall suction at 0–300 mmHg with 11, 12 Fr sheath sizes, varying irrigation pressure IRP was monitored with a Swan–Ganz balloon catheter placed in the calix and connected to a transducer.	Use of SUAS during RIRS can lower mean IRP; however, this effect could reverse with extended suctioning, especially under conditions of high vacuum (>200 mmHg) owing to outflow-tract collapse.

SUAS—suction ureteral access sheath; UAS—(conventional) ureteral access sheath; FANS—flexible and navigable ureteral access sheath; IRP—intrarenal pressure.

### 3.2. Results of Review of Clinical Studies (Table 2, Table 3 and Table 4)

#### 3.2.1. Operative Time

Operative duration in RIRS is an independent risk factor for infectious complications. Consequently, reducing operative time and minimising IRP are essential objectives to ensure surgical safety [39].

SUASs have emerged as a promising solution that could potentially reduce both the operative time and IRP. The first clinical report by Zeng et al. demonstrated a notably short mean operative time of 27.3 min using SUAS, suggesting a potential means to achieve a significant reduction of operative time [40].

Zhu’s comparative study showed that patients undergoing FURS with SUAS had a mean operative time of 49.7 ± 16.3 min, which was significantly shorter than the 57.0 ± 14.0 min observed in the traditional UAS group (*p* < 0.001), indicating the efficiency of SUAS in reducing operative time [41]. However, Qian et al. found no significant difference in operative times between SUAS and traditional UAS groups, with means of 72.9 min and 80.0 min, respectively (*p* = 0.121), demonstrating that the impact of SUAS on operative time may vary depending on other unreported factors [42]. Considering the variability in operative times across studies, the use of SUAS has been enhanced by integrating an intelligent pressure-control system (IPCS). The research by Huang et al. with SUAS and IPCS indicated a notable reduction in operative time in a cohort of 40 patients, with an average of 25.2 min [43]. This finding aligns with Du’s study on the treatment of large ureteral stones below the L4 level, which also reported a significant decrease in operative time using SUAS with IPCS (25.3 min) compared to traditional UAS (47.2 min), *p* < 0.001 [44]. However, Gao’s larger-scale study involving SUAS with IPCS in 278 patients showed an average operative time of 75 min, which again, suggests the influence of unidentified factors [45]. Perhaps factors that limit the ability to remove fragments or debris such as difficult access to parts of the kidney, such as the lower pole, often necessitate the use of accessories like stone baskets, which further add to operative times. A relatively new innovation, whereby to address these factors, the flexible and navigable ureteral access sheath (FANS) with suction, has been upgraded with a flexible distal 10 cm tip, facilitating smoother navigation into each calyx of the pelvicalyceal system. Chen’s initial clinical study demonstrated an average operative time of 70.8 min using 12/14 Fr FANS [46]. In contrast, Gauhar et al. observed a shorter operative duration of 63 min using the same-sized FANS and 76 min with a smaller FANS 10/12CH, which highlights the impact of UAS size on operative time [47]. Zhong’s study, reported an even shorter duration of RIRS using 12/14 Fr FANS for 34.5 min [48]. Huang’s prospective study found a slight reduction in operative time, although statistically insignificant (*p* = 0.235), in time with FANS compared to traditional UAS, averaging 37.7 versus 40.3 min, respectively [49].

**Table 2:** Clinical studies focused on suction ureteral access sheaths (SUAS).

**Author**	**Suction Modality**	**Laser Modality**	**Operative Time**	**Stone Free Rate**	**Definition of Stone Free Rate**
Zeng et al. [40]	First report of SUAS modified from 12/14 Fr UAS (Well Lead Medical, Guangzhou, China) Negative pressure aspirator set to continuous mode at 150–200 mmHg; semirigid scope passes through it	Ho:YAG 0.5–0.6 J 30–35 Hz	27.3 min	97.3% (immediate SFR); 100% (1 month)	no visual stone fragment on fluoroscopy and KUB
Zhu et al. [41]	12/14 Fr SUAS (KYB, Wuxi, China)	Ho:YAG 12–20 W 14–20 Hz	49.7 min (16.3)	82.4%(1 Day), 88.8% (30 Days)	radiological residual <2 mm on KUB or NCCT
Qian et al. [42]	12/14 Fr SUAS (Cook Medical, Bloomington, IN, USA), connected with the negative pressure pump at 0.01 MPa.	Ho:YAG 12–20 W 14–20 Hz	72.9 min (28.1)	Day 1 post-op: 86.4% vs. 71.6%; *p* = 0.034 1-month post-op: 88.9% vs. 82.7%; *p* = 0.368	complete absence of RF or asymptomatic RF < 4 mm at KUB or NCCT at 1 month
Tang et al. [50]	11/13 Fr SUAS (Well Lead Medical, China)	Ho:YAG 200-micron fibre 0.8–1.0 J 15–20 Hz	61.4 ± 5.2 versus 60.3 ± 5.6 (*p* = 0.183)	1 day 73.2% versus 86.2% (*p* = 0.034); 2 week 82.6% versus 90.8% (*p* = 0.110); 4 week 94.2% versus 95.4% (*p* = 0.719)	no radiological evidence of stones or the presence of <= 2 mm asymptomatic fragments on KUB or NCCT
Gauhar et al. [51]	SUAS (ClearPetra, Guangzhou, China) vs. DISS by aspiration	TFL	NR	FANS:66.7% vs. DISS: 64.3%	RF < 4 mm not stone free
Lai et al. [52]	14/16 Fr SUAS (ClearPetra, Well Lead Medical, China)	Ho:YAG 1–1.5 J 15–20 Hz	72.4 (21.3)	14 (50)	absence of any fragments by low-dose NCCT
Wang et al. [53]	35-cm or 25-cm SUAS 12/14 Fr (ClearPetra, China) connected to a vacuum at 30–40 pKa. semirigid scope passes through it. Inflow irrigation with mechanical pump at 60 c.c./minute. The aspiration pressure was set at 200 mmHg.	Ho:YAG 550-micron fibre 0.5–0.6 W 5–30 Hz	33.7 min ± 12.2	33/35 (94.3%)	No stones seen on KUB (immediate postop); No stones on NCCT or KUB (3 months post-op).
**Author**	**Sepsis Rate**	**Ureteric Injury Rate**	**Other Complications**	**Outcome**	
Zeng et al. [40]	Fever 1.9% (Clavien I)	-	Successful SUAS insertion: 77.1% ureteral false passage 0.9% (Clavien IIIa)	Novel technique with modification to the common UAS. Less retropulsion of stone fragments and improved immediate SFR. Continuous irrigation and aspiration yield better visualization and possibly reduce IRP; minimal learning curve and no special equipment required.	
Zhu et al. [41]	5.50%	0	ureteral stricture 0.6%, septic shock 0.6%	Suctioning UAS has advantages of higher SFR one day postoperatively, fewer infectious complications, and shorter operative time.	
Qian et al. [42]	fever: 3.70% vs. 14.8%; *p* = 0.030; SIRS: 1.23% vs. 12.3%; *p* = 0.012)	-	-	The application of suctioning UAS during FURS was associated with higher SFR on day 1 after surgery and a lower incidence of postoperative fever or SIRS.	
Tang et al. [50]	urosepsis: 0% versus 4.6% (*p* = 0.044); fever: 2.4% versus 10.3% (*p* = 0.031)	NR	bleeding: 1.2% versus 3.4% (*p* = 0.317); pain: 2.4% versus 14.9% (*p* = 0.003)	RIRS with SUAS, a new partnership to treat 1–2 cm infectious upper ureteral stones, was satisfying as it achieved a high SFR rate and a low rate of infectious complications. This method was safe and reproducible in clinical practice.	
Gauhar et al. [51]	2 vs. 0	0 vs. 1	Pelvicalyceal system bleeding (7 vs. 1)	Both had high SFR, minimal complications, suction-enhanced vision, 1st comparative study	
Lai et al. [52]	Infection 1 (4); Fever 2 (7)	1 (4)	Steinstrasse: 1(4); Emesis: 1(4)	SUAS improves the stone-free rate in patients with 2–4 cm kidney stones, reducing the comorbidities.	
Wang et al. [53]	Fever—3/35 Sepsis 1/35	0	Haematuria (requiring catheter drainage)—3/35	SUAS in the treatment of complex steinstrasse is safe and effective	

SUAS—suction ureteral access sheath; UAS—(conventional) ureteral access sheath; Ho:YAG—Holmium:Yittrium-Aluminium-Garnet; TFL—Thulium Fibre Laser; KUB—X-ray of kidney ureter and bladder; NCCT—non-contrast computer tomography; RF—residual fragment; IRP—intrarenal pressure; SFR—stone free rate; NR—not reported.

**Table 3:** Clinical studies focused on pressure-sensing regulation.

**Author**	**Accrual Year**	**Country**	**Study Type**	**Groups of Comparison**	**Sample Size**
Huang et al. [43]	Nov 2013–Aug 2015	China	prospective	-	40 patients with solitary kidney
Du et al. [44]	Dec 2014–Jun 2017	China	prospective	negative pressure suctioning system vs. FURS	122
Chen et al. [54]	2014	China	retrospective	FURS with SUAS vs. MPCNL	91
Gao et al. [45]	Jul 2020–Aug 2021	China	retrospective	no	278 patients. 310 kidney units
Deng et al. [55]	Jun 2015–Oct 2020	China	retrospective	FURS with SUAS vs. MPCNL	127 (57 FURS, 70 MPCNL)
**Author**	**Suction Modality**	**Laser Modality**	**Operative Time**	**Stone Free Rate**	**Definition of Stone Free Rate**
Huang et al. [43]	patented irrigation and suctioning platform with UAS	Ho:YAG. 0.8 J 20–30 Hz	25.2 ± 14.5 min	87.5% (4 weeks); 92.5% (12 weeks)	no residual stone and residual stone < 4 mm in size on KUB
Du et al. [44]	SUAS 12/14 Fr 30–45 cm with semirigid URS	Ho:YAG 550 micron fibre 0.6–0.8 J 25–30 Hz	25.3 min (5.6)	100%	residual < 4 mm on KUB
Chen et al. [54]	integrated pressure-measuring SUAS 12/14 Fr	Ho:YAG 0.8 J 30 Hz	65.62 min (22.54)	93.10%	residual < 3 mm on KUB
Gao et al. [45]	patented irrigation and suctioning platform with UAS	Ho:YAG 0.8–1.6 J 20–30 Hz	75 min (60–110 min)	One-session SFR and one-month SFR were 80.65% and 82.26%.	no residual stone or residual stone < 4 mm in size by KUB
Deng et al. [55]	UAS 12/14 Fr with pressure measuring and suctioning	Ho:YAG 0.8 J 20 Hz	61.8 ± 21.1 min SUAS group. 43.4 ± 18.9 min MPCNL group	3 m 91.2 (52/57) SUAS group. 95.7 (67/70)MPCNL Group	<2 mm on NCCT
**Author**	**Sepsis Rate**	**Ureteric Injury Rate**	**Other Complications**	**Outcome**	
Huang et al. [43]	fever 5%	-	-	intelligently pressure-controlled flexible URS in treating upper urinary tract calculi for patients with a solitary kidney with advantages of high lithotripsy efficacy and low complication rate.	
Du et al. [44]	1.60%	0	-	treatment of large urinary tract stone >1.5 cm with system shows shorter operative time, lower incidence of postoperative fever and secondary surgery, and higher stone clearance rate	
Chen et al. [54]	10.80%	0	-	for single kidney stone with a diameter of 2–3 cm, intelligent pressure-controlled FURS and MPCNL are both effective treatment methods, but the FURS has advantages, such as fewer complications, shorter hospital stay, and less bleeding.	
Gao et al. [45]	nil	-	CD reporting. 8 patients had Clavien–Dindo Grade II, and 2 patients had Grade III complications (ureter lesions)	good safety and efficacy of patented irrigation and suctioning platform with UAS, with one-session SFR of 80.65% (250/310) and a low complication rate (3.26%). Patients with stone size < 40 mm or Guy’s stone score of Grade I had a significantly higher potential to reach stone-free after treatment.	
Deng et al. [55]	SUAS Group 5 (8.8%). MPCNL group 6 (8.6%)	-	Ascites SUAS 1 (1.8%) MPCNL 2 (2.9%)	FURS with SUAS and MPCL with 2–3 cm renal stones in solitary kidneys are effective. MPCNL shorter operative time, FURS with SUAS has less bleeding, shorter hospital stay and less damage to kidney function	

SUAS—suction ureteral access sheath; UAS—(conventional) ureteral access sheath; MPCNL—minimally invasive percutaneous nephrolithotomy; Ho:YAG—Holmium:Yittrium-Aluminium-Garnet; KUB—X-ray of kidney ureter and bladder; NCCT—non-contrast computer tomography.

**Table 4:** Clinical studies focused on flexible and navigable ureteral access sheath (FANS).

**Author**	**Accrual Year**	**Country**	**Study Type**	**Groups of Comparison**	**Sample Size**
Chen et al. [46]	Aug 2021–Jan 2022	China	prospective	-	53
Gauhar et al. [47]	Nov 2021–Oct 2022	Multi-centre	retrospective	FANS 10/12 vs. 12/14	35
Zhong et al. [48]	Jun 2016–Jan 2018	China	retrospective	No	52
Huang et al. [49]	Feb 2022–Feb 2023	China	retrospective	Yes	371
Chen et al. [56]	Jan 2022–Nov 2022	China	retrospective	FURS with FANS versus miniPCNL	96 pts FURS with FANS versus 96 miniPCNL
Zhang et al. [57]	Aug 2021–Apr 2022	China	retrospective	FANS vs. UAS	214 pts (102 FANS versus 112 UAS)
Liang et al. [58]	Oct 2021–Nov 2022	China	retrospective	no	224
Yu et al. [59]	Jan 2021–Sep 2022	China	retrospective, matched-pair analysis	FANS vs. UAS	FANS 152/conventional 152
Wang et al. [60]	Jul 2017–Jul 2018	China	retrospective	vacuum UAS vs. miniPCNL	28 vs. 56
Gauhar et al. [61]	Sep 2022–Mar 2023	Multi-centre	retrospective	No	45
**Author**	**Suction Modality**	**Laser Modality**	**Operative Time**	**Stone Free Rate**	**Definition of Stone Free Rate**
Chen et al. [46]	FANS (Zhangjiagang, China) 12/14 Fr female: 36 cm; male: 46 cm (negative pressure: 50–150 cmH_2_O)	Ho:YAG 20–40 Hz 0.6–1.2 J	70.8 (26.9)	29 (69.8%)	stone volume clearance rate = 1 − (residual stone volume/preoperative stone volume) × 100%
Gauhar et al. [47]	FANS Elephant II first or second generation (Zhejiang YiGao Medical Technology Co., Ltd., Hangzhou, China) 10/12 Fr and 12/14 Fr 40 to 55 cm (negative pressure: 0.02 MPa)	TFL 0.2–0.4 J 200–400 Hz and HP Ho:YAG 0.4 J 40 Hz	76 vs. 63 min	94.7% vs. 68.8%	Stone-free status was defined as the absence of a single RF > 2 mm on NCCT.
Zhong et al. [48]	Flexible pressure-measuring ureteroscopic sheath 12–14 Fr	Ho:YAG	34.5 ± 18.3 min	95.7% at Day 1–2, 100% at one month	Residual stone < 4 mm at KUB X-ray or CT-scan if X-ray was negative
Huang et al. [49]	FANS 11/13 or 12/14 Fr	Ho:YAG	40.3 ± 18.9 min in traditional FURS group, 37.7 ± 20.1 min in suction group	52 (50.5%) and 81 (78.6%) in traditional FURS and suction group respectively at 1 day, 78 (75.7%) and 97 (94.2%) in traditional FURS and suction group respectively at 30 days	Residual fragments < 3 mm at CT scan
Chen et al. [56]	12/14 Fr, 36 cm for female, 46 cm for males, FANS (Woek, Nanchang, China): negative pressure value to 2–7 Kpa. The irrigation volume was adjusted to a range of 80–200 mL/min.	Ho:YAG 1.0–1.2 J 15–30 Hz.	49.3 (11.9), 25–74 versus 50.6 (11.4), 25–71 (*p* = 0.06)	85.4% versus 90.6% (0.266)	NCCT showing zero stone fragments
Zhang et al. [57]	12/14 Fr UAS (Shenzhen Kang Yi Bo Technology Development Co., Ltd., Shenzhen, China) 45 for male 35 for female; FANS 12/14 Fr (Zhangjiagang Huamei MedicalEquipment Co., Ltd., Zhangjiagang, China) 45 cm for male 35 cm for female negative pressure was set at −20 to −60 kPa.	Ho:YAG 0.6–1.2 J 5–20 Hz for fragmentation, and the dusting mode using 0.2–0.6 J 20–30 Hz.		1 d 86.3% versus 75.0% (*p* = 0.038); 30 d 91.2% versus 81.3% (*p* = 0.037)	no residual stone or radiological residue fragment < 2 mm
Liang et al. [58]	FANS 12/14 Fr (Elephant II, Zhejiang YiGao Medical Technology Co., Ltd., Hangzhou, China)	Ho:YAG 1–1.5 J 15–20 Hz.	69.2 ± 65.2 min	postoperative day 1: 172/224 (76.8%); postoperative day 30: 218/224 (97.3%);	absence of any stones or residual fragments ≤ 2 mm under non-contrast CT
Yu et al. [59]	FANS–12/14 Fr; Zhangjiagang, China. Conventional UAS–12/14 Fr; Zhangjiagang, China	Ho:YAG 1.0–1.2 J; 15–30 Hz	FANS 56.5 ± 13.9/UAS 59.9 ± 16.2	FANS 116 (76.3%) UAS 11 (7.2%) *p* < 0.001) at 1 day postoperatively No difference at 1 month	zero stone fragments at CT scans on 1st day and 1 month after the surgery.
Wang et al. [60]	FANS (ClearPetra, Well Lead Medical, China)	Ho:YAG	RIRS 72.4 (21.3), 42–106 miniPCNL 67.4 (25), 44–114	RIRS 25 (89.3%) miniPCNL 52 (92.9%)	zero fragments on low-dose CT on postoperative day 1.
Gauhar et al. [61]	FANS Clearpetra 12/14 Fr	Not mentioned	65 min	93.3% at three months	Absence of stone fragments at CT scan
**Author**	**Sepsis Rate**	**Ureteric Injury Rate**	**Other Complications**	**Outcome**	
Chen et al. [46]	2 (4)	0	Emesis: 2 (3.8%)	intelligently pressure-controlled flexible URS in treating upper urinary tract calculi for patients with a solitary kidney with advantages of high lithotripsy efficacy and low complication rate.	
Gauhar et al. [47]	4 vs. 0	0	1 fornix rupture	treatment of large urinary tract stone > 1.5 cm with system shows shorter operative time, lower incidence of postoperative fever and secondary surgery, and higher stone clearance rate	
Zhong et al. [48]	Fever 1/52	1 Ureteral extravasation	8/52 Haematuria without transfusions	Ureteroscopic lithotripsy with intelligent pressure control improves the efficiency of the lithotripsy and rate of stone clearance	
Huang et al. [49]	Fever rate: 3.6% vs. 6.3% in traditional FURS and suction groups, respectively. Absence of sepsis	Non-reported	Not reported	vacuum-assisted dedusting lithotripsy (VADL) technique can significantly improve the postoperative SFR for the patients with kidney or proximal ureteral stones less than 3 cm in size treated by flexible ureteroscope.	
Chen et al. [56]	infection: 0% versus 3.1%; fever 4.2% versus 7.3%	NR	total complications: 5.2% versus 13.5% (*p* = 0.048); emesis: 1% versus 4.2%; transfusion: 0% versus 1%; interventional embolization 0% versus 1%	In the treatment of 2–3 cm renal stones, FURS with a novel FANS may provide a superior alternative to mini-PCNL, potentially challenging its established status	
Zhang et al. [57]	infectious: 8.8% versus 18.8% (*p* = 0.037) (fever: 3.9% versus 9.8%) (urosepsis 3.9% versus 6.3%) (septic shock 1% versus 2.7%)	0 versus 0	overall complications: 11.8% versus 22.3% (*p* = 0.041); Hb loss −0.54 +/− 0.69 g/dl versus −0.83 +/− 0.66 g/dl, *p* = 0.002); steinstrasse: 0% versus 1.8%	Compared to UAS combined with flexible ureteroscope for treating unilateral renal calculi, FANS had superiority in higher SFR on 1 day and 30 days postoperatively. Shorter operation time, lower haemoglobin loss, and lower incidences of overall and infectious CR were observed in FANS group.	
Liang et al. [58]	Fever–2/224	0	0	for single kidney stone with a diameter of 2–3 cm, intelligent pressure-controlled FURS and MPCNL are both effective treatment methods, but the FURS has advantages, such as fewer complications, shorter hospital stay, and less bleeding.	
Yu et al. [59]	FANS 9 (5.9%)/conv UAS 28 (11.9%)	no	no	FANS has a higher SFR 1 day postoperatively. In addition, FANS has contributed to shorter operative time and fewer complications.	
Wang et al. [60]	FANS-2. miniPCNL-5	no	no	In the treatment of 2–4 cm renal stone, using FANS in RIRS can improve surgical efficiency with lower postoperative early pain scores.	
Gauhar et al. [61]	Fever 16/45 (35.6%), Sepsis 0/45	3/45 (6.7%)	Reintervention for residual fragments 3/45	FANS improves single-session SFR and reduces the need for a ureteric stent or catheter	

UAS—(conventional) ureteral access sheath; MPCNL—minimally invasive percutaneous nephrolithotomy; Ho:YAG—Holmium:Yittrium-Aluminium-Garnet; TFL—Thulium Fibre Laser; KUB—X-ray of kidney ureter and bladder; NCCT—non-contrast computer tomography; RF—residual fragment; SFR—stone-free rate.

#### 3.2.2. Stone Free Rates

The definition of SFR was very heterogeneous over our cohort of studies. There were seven SUAS clinical studies. Zhu et al. [41] and Tang et al. [50] deemed SFR as no radiological residual seen or <2 mm on plain abdominal KUB (KUB) or non-contrast computed tomography (NCCT). Qian et al. [42] and Gauhar et al. [51] defined SFR as the asymptomatic RF < 4 mm on KUB or NCCT, whereas Lai et al. [52] and Wang et al. [53], by contrast, determined SFR as a complete absence of fragments on NCCT. Despite these varied definitions, the general SFR ranged from 71% to 97.3% across most studies. The lowest SFR reported was by Gauhar et al. [51], at 64.3%, but it must be noted that it was the only study that utilized TFL as opposed to a Ho:YAG laser. The most significant difference noted in SFR was in Qian et al. [42], where they retrospectively evaluated SUAS compared to UAS. Although the postoperative SFR difference was greatest on day 1 SFR (86.4% vs. 71.6%), it was comparable and not statistically significant at 1 month postoperatively (88.9% vs. 82.7%, *p* = 0.368).

There were five clinical studies that included SUAS with a pressure-regulating system. Interestingly, none defined SFR as no residual fragments but rather mainly < 4 mm on KUB [43,44,45,54], with only one study as <2 mm on NCCT [55]. Comparatively, the overall SFR ranged higher in the SUAS group, from 87.5% upwards. All of these studies utilized a Ho:YAG laser. Only one study by Du et al. reported a 100% SFR [44], defined as residual < 4 mm on KUB, which demonstrated that the use of their patented perfusion and suctioning platform was effective and safe with better SFR.

Lastly, there were 10 studies with FANS which were mainly reported in the recent 3 years. Indeed, this is a true reflection of how technological advancements in UAS are rapidly influencing evidence generation and reiterating the need for a timely update, like in this review. Most studies had similar definitions of SFR as above, with Chen et al. [56] using the stone volume clearance rate instead. Of note, the most significant difference in SFR reported among the studies was Gauhar et al. [47], where the size of FANS was compared (10 Fr vs. 12 Fr), with a significantly higher SFR in the 10 Fr group (94.7% vs. 68.8%, *p* = 0.01). The other trend noted among the studies was the significant change in SFR reported on postoperative day 1 compared to 30 days. Zhang et al. [57] evaluated FANS against UAS and showed that the FANS group had a higher SFR compared to the UAS group on both postoperative day 1 (86.3% vs. 75.0%; *p* = 0.038) and day 30 (91.2% vs. 81.3%; *p* = 0.037). Huang et al. [49] similarly reported a significant change in SFR at day 1 (78.6% vs. 50.5%, *p* < 0.001) compared to day 30 (94.2%% vs. 75.7%, *p* < 0.001), when they evaluated RIRS with FANS vs. without FANS.

#### 3.2.3. Complications

In an initial experience with SUAS reported by Zeng et al. [40], among 104 consecutive patients undergoing ureteroscopy for ureteral stones, only a few complications were observed, such as two cases of fever and one minor ureteral false passage. Zhu et al. [41] compared cases undergoing FURS with SUAS versus traditional UAS for renal stones and found a significantly lower incidence of overall complications, fever, and urosepsis in the SUAS group (11.5% vs. 24.8%, *p* < 0.001; 5.5% vs. 13.9%, *p* = 0.009; 1.8% vs. 6.7%; *p* = 0.029, respectively), yet no significant differences were noted for septic shock, haematuria, steinstrasse or ureteral stricture. Similarly, Qian et al. [42] evaluated patients undergoing FURS with FANS and traditional UAS and reported a lower incidence of postoperative fever or systemic inflammatory response syndrome (SIRS) in the SUAS than the traditional UAS group (fever 3.7% versus 14.8%, and SIRS 1.2% versus 12.3%; both *p* = 0.03). Lai et al. [52], retrospectively comparing RIRS with SUAS versus minimally invasive percutaneous nephrolithotomy(MPCNL) for the treatment of 2–4 cm renal stones, found a lower overall complication rate in the RIRS group, yet the difference was not statistically significant (21.4% versus 23.2%; *p* = 0.854). More specifically, postoperative fever and urosepsis were encountered in two and one cases in the RIRS group, and five and two patients in the MPCNL group, respectively. Additionally, one ureteral perforation occurred in each group, blood transfusion was required for two patients in the MPCNL group, and one patient in the RIRS group developed steinstrasse. On the contrary, in the study by Tang et al. [50], which included patients with 1–2 cm infectious upper ureteral stones who were randomly assigned to RIRS with SUAS or MPCNL, postoperative complications were significantly lower in the RIRS than the MPCNL group with regards to fever, pain, and urosepsis (2.4% vs. 10.3%, *p* = 0.031; 2.4% vs. 14.9%, *p* = 0.003; 0 vs. 4.6%, *p* = 0.044; respectively). However, no significant difference was found in bleeding between the two groups (1.2% vs. 3.4%, *p* = 0.317). SUAS was also evaluated for the treatment of complex steinstrasse. In a recent prospective case series including 35 patients, three cases of fever, including one with chill and transient hypotension, and three with significant haematuria were reported [53]. Interestingly, Gauhar et al. [51], reporting on a comparison among three different techniques of suction in RIRS, namely SUAS, direct in-scope suction (DISS), and FANS, found no major intra- or postoperative complications for all the methods. More specifically, the authors observed fever and sepsis (Clavien–Dindo grade I) in 36.7% and 6.7% of cases using scope suction and 25% and 0% for SUAS, respectively.

With specific regard to SUAS with automatic control of IRP, Huang et al. [43], among a cohort of 40 patients with upper urinary tract stones and a solitary kidney, reported only two cases of postoperative fevers, and no other complications were observed. Similarly, Du et al. [44] in their study on patients with large ureteral stones below the L4 level randomly assigned to RIRS with SUAS monitoring IRP vs. those with traditional UAS, reported fewer cases of postoperative fever (one versus seven, *p* = 0.03), with no cases of ureteral stricture or perforation. A low Clavien–Dindo II-III complication rate (3.26%) was also found in the retrospective cohort by Gao et al. [45] using FURS and SUAS with intelligent pressure control for upper urinary tract stone treatment. Chen et al. [54], evaluating FURL using SUAS with intelligent control of RPP versus MPCNL in patients with 2–3 cm renal stones, found a lower overall complication rate in the first groups (11.3% versus 28.8%, *p* = 0.03), yet differences were not significant for individual complications, such as fever (three patients for each group). More recently these two approaches were also evaluated in the same setting of patients—yet having a solitary kidney—and the authors found no significant differences in complications—except for blood transfusion, which was higher in the MPCNL group (0 versus 10%, *p* = 0.016) Of note, fever was observed in 8.8% versus 8.6% of patients (*p* = 0.9), respectively [55].

Various complications in the use of FANS have been reported. Chen et al. [46] reported only two cases of fever in a cohort of 53 patients who underwent FURS with FANS, while Liang et al. [58] recorded an even lower fever rate (2/224). In another study, 10/12 Fr FANS have been compared to 12/14 Fr, reporting four cases of fever for the first group and no infectious complications for the second group [47]. Some studies have compared FANS to traditional UAS. Zhang et al. [57] reported a lower infection and fever rate (8.8% vs. 18.8%, *p* = 0.037 with 3.9% vs. 6.3% urosepsis, respectively) for FURS with FANS compared to traditional UAS. Similar results were recorded by Yu et al. [59], with a fever rate of 5.9% in the FANS group vs. 11.9% in the traditional UAS group, and by Huang et al. [49], with a fever rate of 2.9% for traditional UAS vs. 3.9% for FANS. Chen et al. [56] compared FURS with FANS vs. MPCNL, reporting a lower rate of overall complications and infection for patients undergoing FURS (5.2% vs. 13.5% for complications and 0% vs. 3.1% for infections). Wang et al. [60] also showed a lower fever rate for the FANS group compared to PCNL (2/28 vs. 5/56, respectively). Cases of ureteral damage using FANS have been described by Zhong et al. [48] and Gauhar et al. [61] (1.9% and 6.7%, respectively).

## 4. Discussion

### 4.1. History of Development of the UAS (Figure 2)

Quoting Arthur Clarke, “any sufficiently advanced technology is indistinguishable from magic”. Indeed, in endourology, we see that over the decades, scientific, technical and technological advancements have magically transformed the way modern flexible ureteroscopy (FURS) is performed. Along with advancements in lasers, miniaturisation, and digitalisation of scopes, accessories in FURS too have undergone a transformational change to improve RIRS outcomes. In our study, we share the historical development of the UAS over the decades and its current role in FURS based on a review of experimental and clinical studies.

Whilst evaluating the first successful ureteroscopic evaluations of the upper urinary tract, Takayasu and Aso noted the insertion of the scope into the ureter was a hurdle to overcome. In 1974, they reported the concept of a “guide tube” made of Teflon that allowed the passage of the ureteroscope to the upper tract. This was the predecessor of the modern ureteral access sheath [1]. Subsequently, after a decade, Newman et al. described a novel ureteral access sheath–dilator system in 1985 that was used in a series of 43 procedures over 18 months and reported a 51% stone-free rate when used for FURS, a 92% rate of successful ureteral stricture dilation, and an 88% success rate of diagnostic evaluation of upper tract filling defects [62]. Yet they also had an 18% incidence of UAS-induced ureteral perforations. In 1987, the “peel-away” introducer sheath was first reported by Rich et al. The peel-away introducer sheath set was actually meant to dilate a percutaneous nephrostomy tract and establish a temporary access conduit for manipulation in the ureter. This was a 60 cm sheath available in 8 to 18 Fr and packaged with a 0.038-inch 145 cm stainless-steel guidewire and a radiopaque 65 cm polyethylene introducer 2 Fr polytetrafluoroethylene (Teflon) sheath was successfully used by the team for retrograde silicon stent placements, stone extraction, and catheterisation of tortuous ureters. Its soft material and successful deployment in a retrograde fashion allowed them to successfully use the 11 Fr peel-away sheath as a working sheath for the 9 Fr flexible ureteroscope to examine the ureter after stone removal and to remove any missed stone fragments with a basket if needed. Later, they deployed larger peel-away sheaths (14 Fr) in all cases of difficult ureteral access to deploy flexible instruments. Their study concluded that this sheath was very useful, especially when multiple ureteral access was needed with FURS [63]. The Finlayson ureteral dilator–sheathing system, which consists of coaxial Teflon catheters from 6, 10, 14, and 17 French was introduced to solve the problem of ureteral mucosal interposition between individual sheaths. In their initial reports, the authors used a 23.5 Fr Storz panendoscope to visualise the ureteral orifice in question and then fluoroscopically deployed the system. They reported that this facilitated the easy passage of an 11.5 Fr Storz ureteroscope, which was then the standard semirigid ureteroscope, allowing for basketing and ureteral stone removal. By providing a conduit for ureteral access that allows for inspection of the ureter and the calyceal system, it was then used with a flexible endoscope [64]. Quoting that, despite advances in being able to use the ureteral dilator technique and devices to access the ureter and renal system for FURS, ureteral perforation occurred in 6% of the patients; hence, in 2001 Kourambas et al. reported using the 12/14 Fr UAS that had an impregnated wire and hydrophilic coating, facilitating safer and direct visual insertion of the flexible scope rather than railroading over a wire. The authors randomized 59 patients to semirigid or flexible ureteroscopy with or without a UAS and cited that routine use of a UAS was associated with decreased operative time without an increase in the complication rate, advocating its routine use [65]. Importantly, this study in the ensuing years paved the way for additional studies on these devices focused on how these helped decrease IRP during ureteroscopy, which had a positive correlation with postoperative pain and infection. Furthermore, using these also increased the time between repairs of digital flexible ureteroscopes by minimizing ureteroscope damage [66,67,68]. In a worldwide survey by the Endourological Society in 2015, the practice of using UAS, whilst not a standard recommendation in the guidelines, was preferred by 58% of surgeons [69].

In 2015, Liu et al. [70] were the first to design a patented intelligent system to facilitate lithotripsy efficiency that included an irrigation and suctioning platform and an 11.5–15 Fr UAS with a pressure-sensitive tip. It had two connecting channels on the back end of the UAS, which were connected to the suction vacuum device and pressure monitoring feedback devices, enabling precise fluid inflow regulation and control of the vacuum suctioning by computerised real-time recording and monitoring of IRP, thus heralding a new era of the use of suction in FURS. Zeng et al. [40] were the first to report a simple modification to the standard UAS in 2016. The modified 12–14 Fr UAS had an oblique suction-evacuation port with a pressure vent at the distal end of a traditional UAS that had a pressure-regulating mechanism to allow active egress of irrigation fluid and stone fragments. In the 104 patients that were enrolled, they reported that the device helped provide safe and effective treatment for the majority of the ureteral stones and likely reduced the risk of retropulsion of the stone fragments from the ureter and improved the immediate SFR. With continuous irrigation and aspiration, they also noticed clearer visualization throughout the procedure. They acknowledged that this had a short but easy learning curve, and it was imperative to ensure adequate irrigation flow. Huang J. et al. [43] introduced the patented irrigation and suctioning platform that allowed users to choose between four models of automatic (perfusion, suctioning, pressure monitoring, and pressure feedback control), semi-automatic (pressure monitoring, and perfusion), pure perfusion, and pure suctioning, respectively, tailoring to the operative requirements. The advantage was that for the first time, the platform monitored IRP that could be displayed in real time by virtue of the two connecting channels on the back end of the UAS that were connected to the suction vacuum device and pressure-monitoring feedback device. They succeeded in utilizing this platform in 40 patients with solitary kidneys for upper urinary tract calculi with minimal complications.

As further studies explored the utility of suction ureteral access sheath (SUAS), the utility of suction in endourology found a firm footing, as was reported by Quhal et al. [29]. By deploying suction and aspiration, urologists could facilitate stone debris removal, reduce IRP, allow for and potentially decrease the operation time and infectious complications, and improve SFR. Yet, one limitation of the SUAS was a challenge in removing all the debris and dust, especially in dependent calyces. In 2022, Chen Y. et al. reported the first use of a novel vacuum-assisted flexible and navigable ureteral access sheath (FANS) versus the traditional ureteral access sheath (UAS) in an experimental porcine model in simulating RIRS [35]. The reported FANS exhibited good flexibility and deformability at the tip when used with a flexible scope bending up to 90° without a scope and reportedly up to 145° with a flexible ureteroscope. Additionally, it was reinforced with wire springs to ensure that the lumen did not collapse on bending and could be connected to a suction device. They called this the vacuum cleaner in the kidney and were able to show that the FANS can be placed across the ureteropelvic junction and directed into the renal pelvis and calyces. It could actively reduce the IRP to the desired range by adjusting the negative value under any irrigation fluid velocity. Navigating the FANS close to the stone can achieve complete stone-free status in RIRS.

The first clinical study of FANS was done by Gauhar et al., who reported on the ability to use a scope to navigate the proximal flexible 10 cm of the sheath into the desired calyx both by active and passive deflection, accentuating the ability to remove dust and debris even in dependent calyces like lower pole. The authors coined the term FANS to standardise the use of any vacuum-assisted flexible and navigable ureteral access sheath and quoted that, if replicated in other studies, it could be a potential game changer [47]. They also dedicated a step-by-step video to train surgeons on how to use FANS [61]. With the successful advent of FANS, it has been modified by different companies. Recently in 2023, a new modification reportedly called omnidirectional UAS (ODUAS) made of PEBAX (polyether block amide) with the whole sheath well supported by a metal-wire coil to prevent its collapse under pressure was used in 199 patients. The intraluminal channel is coated with Teflon (polytetrafluoroethylene) and the outside layer is coated with hydrophilic polyvinylpyrrolidone, which makes it less atraumatic at insertion and ureter friendly. The 10 cm of the tip are flexible and the last 3 mm are soft and collapsible, making it atraumatic. The suction port is modified, consisting of a nozzle, a suction switch, and a watertight valve allowing for a better-improved experience. Despite these modifications, the principles of use are the same as that for FANS. The authors also suggest that ODUAS by YiGao Medical was comparatively better and easier to insert and place. Its benefit includes that it could shorten the UAS tip–stone distance, thus improving fragments suction, substantially expediting the lithotripsy process, and even expanding the indication of RIRS in terms of stone size. In their retrospective study, stones that were >2 cm and <3 cm were generally cleared within 1 h in a single session.

**Figure 2:**
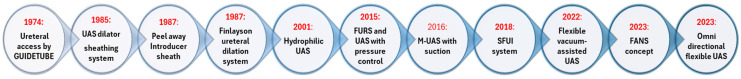
Evolution of ureteral access sheath in RIRS.

### 4.2. The Influence of Pressure with the Use of SUAS and FANS in RIRS

The normal physiological intrarenal pressure is approximately 10 mmHg [71]. The threshold for pyelovenous and pyelosinous backflow has been shown to be 30–45 mmHg [72]. The intrarenal pressure can be dramatically increased up to more than 300 mmHg during retrograde intrarenal surgery, raising concerns for increased risk of sepsis [7,72,73,74,75,76]. Studies have proven that the use of UAS can prevent the elevation of intrarenal pressure [66,67,77,78].

Whitaker laid the foundations of the antegrade pressure measurement of the upper urinary tract—the Whitaker test [79,80]. Typically, IRP was measured by a percutaneously placed pressure transducer catheter in the renal pelvis or a pre-existing nephrostomy tube in both animal and clinical studies [81,82,83,84]. It was only in 2008 that retrograde ureteral catheters were innovatively used to record IRP. The first report was on the placement of a ureteral catheter at the renal pelvis connected to a transducer [85], followed by studies on a SUAS with a pressure-measuring function [36,43,44,45,48,70,86], and studies on the off-label utilisation of a pressure-sensor flexible wire commonly used in cardiovascular interventions [82,87,88,89,90,91,92].

With the evolution of suction application in retrograde intrarenal surgery and the advancing technologies that allow for IRP measurement and a better physiological understanding of the detrimental effects of high IRP, the use of SUAS and FANS in RIRS has gained momentum to prevent the complications related to high IRP and temperature [27,93,94,95,96].

Real-time monitoring of pressure profiles with the use of suction UAS, as reported in aforementioned studies, is changing the way urologists are able to perform RIRS even for bigger stones by not only improving the laser efficiency of high-power lasers but almost mitigating septic complications even when using a smaller diameter UAS compared to an older larger diameter traditional UAS [91,97]. This was proposed by Lildal et al. [98] and proven by Jahrreis et al. [99].

### 4.3. Future Direction

**Figure 3:**
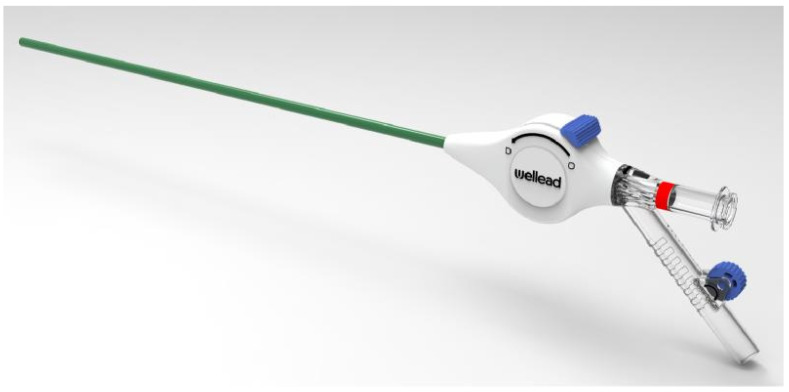
Second generation of suction ureteral access sheaths (permission granted from Well Lead Medical Co., Ltd., Guangzhou, China).

**Figure 4:**
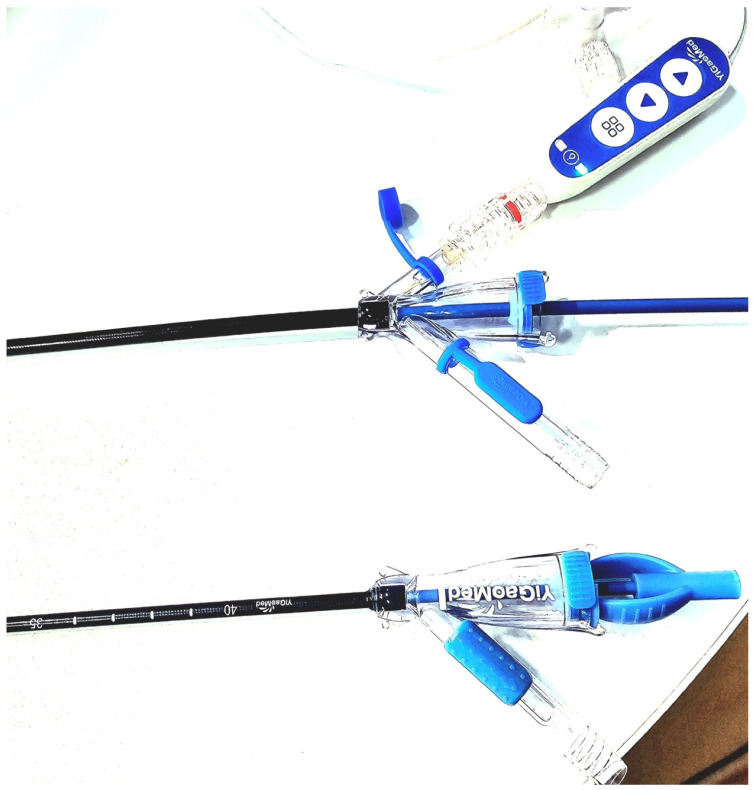
Newer generation of suction ureteral access sheaths with pressure monitoring and regulatory system (permission granted from YiGao Medical, Zhejiang, China).

The suction-assisted ureteral access sheath with intelligent pressure systems will enable surgeons to push the boundaries of RIRS, allowing for the successful management of complex cases, stones in the lower pole, and potentially even larger stones [31,52,57,100]. The future evolution of the FANS design may incorporate better steerability designed to lock into specific renal calyces for easy access and allow for precise vent-controlled pressure regulation and ergonomic rotational movements to minimise scope stress [100] (Figure 3 and Figure 4).

Standardised suction values regarding optimal suction levels are needed in practice, especially when using FANS to avoid inadvertent tissue trauma. This was highlighted by Jahrreiss et al. [99].

Akin to the experimental evidence shown earlier by Miguel et al. [101], it remains to be explored to identify the best cross-section and orientation of a ureteroscope within a FANS to achieve the lowest IRP and maximise outflow [22].

As we move towards miniaturisation in endourology, particularly flexible ureteroscopes, it is important to understand the best combination of scope and FANS sizes. As of now Gauhar et al. propose that 7.5 Fr and 10/12 Fr FANS may be the ideal combination, even in non-stented ureters [47]. Perhaps with the further miniaturisation of flexible ureteroscopes, there may be a day when RIRS becomes an outpatient office-based procedure under local anaesthesia using SUAS [102,103].

The biggest question that remains to be answered is this: will FANS and the subsequent newer generation SUAS introduce a preferential boss to use access sheaths or will other suction technologies that incorporate direct in-scope suction with pressure control become the next stand? More studies need to be conducted with the evolution and advancements in technology and techniques to bring about new standards and optimise surgical outcomes.

## 5. Conclusions

Our review highlights how technological modification of the UAS is modifying surgical intervention by RIRS. With SUAS and FANS showing definite promise in minimising infectious complications and significantly improving SFR, it remains to ascertain if the pendulum is moving in favour of using UAS again. Indeed, as we incorporate suction and pressure management and UAS in RIRS, it will definitely improve the outcomes of FURS. Future studies that compare DISS with FANS and SUAS can determine how, when, and where to use which type of intervention. Lastly, our review highlights the urgent necessity that, with evolution, it is time to standardise the outcome reporting in RIRS with suction technologies.

## Data Availability

The data that support the findings of this study are available from the corresponding author upon reasonable request.
